# Is there a place for mesenchymal stromal cell-based therapies in the therapeutic armamentarium against COVID-19?

**DOI:** 10.1186/s13287-021-02502-7

**Published:** 2021-07-27

**Authors:** Kátia Nunes da Silva, André Luiz Nunes Gobatto, Zaquer Suzana Munhoz Costa-Ferro, Bruno Raphael Ribeiro Cavalcante, Alex Cleber Improta Caria, Luciana Souza de Aragão França, Carolina Kymie Vasques Nonaka, Fernanda de Macêdo Lima, Miquéias Lopes-Pacheco, Patricia Rieken Macêdo Rocco, Bruno Solano de Freitas Souza

**Affiliations:** 1 Goncalo Moniz Institute, Oswaldo Cruz Foundation (FIOCRUZ), Rua Waldemar Falcão, 121, Candeal, Salvador, Bahia 40296-710 Brazil; 2 D’Or Institute for Research and Education (IDOR), Salvador, Brazil; 3 Center for Biotechnology and Cell Therapy, São Rafael Hospital, Salvador, Brazil; 4 Graduate Program in Medicine and Health, Faculty of Medicine, Federal University of Bahia, Salvador, Brazil; 5 Laboratory of Pulmonary Investigation, Carlos Chagas Filho Institute of Biophysics, Federal University of Rio de Janeiro, Rio de Janeiro, Brazil; 6 National Institute of Science and Technology for Regenerative Medicine, Rio de Janeiro, Rio de Janeiro, Brazil; 7 COVID-19 Virus Network, Ministry of Science and Technology, and Innovation, Rio de Janeiro, Brazil

**Keywords:** COVID-19, Cell therapy, Mesenchymal stromal cells, Acute respiratory distress syndrome, SARS-CoV-2

## Abstract

The COVID-19 pandemic, caused by the rapid global spread of the novel coronavirus (SARS-CoV-2), has caused healthcare systems to collapse and led to hundreds of thousands of deaths. The clinical spectrum of COVID-19 is not only limited to local pneumonia but also represents multiple organ involvement, with potential for systemic complications. One year after the pandemic, pathophysiological knowledge has evolved, and many therapeutic advances have occurred, but mortality rates are still elevated in severe/critical COVID-19 cases. Mesenchymal stromal cells (MSCs) can exert immunomodulatory, antiviral, and pro-regenerative paracrine/endocrine actions and are therefore promising candidates for MSC-based therapies. In this review, we discuss the rationale for MSC-based therapies based on currently available preclinical and clinical evidence of safety, potential efficacy, and mechanisms of action. Finally, we present a critical analysis of the risks, limitations, challenges, and opportunities that place MSC-based products as a therapeutic strategy that may complement the current arsenal against COVID-19 and reduce the pandemic’s unmet medical needs.

## Introduction

Coronavirus disease 2019 (COVID-19) originated in Wuhan, China, and spread rapidly to pandemic levels, resulting in high morbidity and mortality and causing healthcare systems to collapse worldwide. The causative agent, severe acute respiratory syndrome coronavirus 2 (SARS-CoV-2), is a positive-sense, single-stranded RNA virus of the family Coronaviridae. The virus is transmitted among humans, mainly through respiratory droplets [1]. Most infected individuals remain asymptomatic or have mild respiratory tract symptoms; moderate to severe cases with pneumonia and acute respiratory distress syndrome (ARDS) are associated with the need for long-term hospitalization in intensive care units, prolonged ventilatory assistance, high mortality rates, and potential long-term morbidity in survivors [2].

Although immune responses are critical in controlling and eradicating viral infections, SARS-CoV-2 has been shown to induce an exacerbated inflammatory response, which plays a central role in the pathogenesis and progression of severe COVID-19 [3]. The implications of combined inflammatory and viral-mediated damage may extend to multiple organ dysfunction syndrome [4, 5]. Since the early phase of the pandemic, different strategies have been investigated for the treatment of COVID-19 pneumonia, including antiviral agents, antibiotics, anticoagulants, and immunomodulatory agents, among others [5, 6]. However, only a few of these studies have consistently shown a clinical benefit, as shown for dexamethasone, which significantly decreased 28-day mortality among hospitalized patients requiring supplemental oxygen [7]. The use of the antiviral remdesivir was also associated with a decreased length of hospital stay, although no significant effects were found in terms of reducing mortality [8]. Based on the finding of elevated interleukin-6 (IL-6) serum levels in COVID-19 patients—associated with disease severity and mortality—tocilizumab and sarilumab, recombinant human monoclonal antibody IL-6Rα antagonists, have also been considered, with conflicting results [9–19]. Recently, the results of the RECOVERY trial, which included 4116 adults with severe COVID-19 at 131 sites in the UK, showed reduced mortality and invasive mechanical ventilation requirements [17].

In addition to pharmacological therapy, immunization strategies targeting prevention or early treatment have proven to be crucial. Passive immunization through infusion of convalescent plasma or monoclonal antibodies has demonstrated the potential to treat the disease, especially if applied early [20–23]. Now that vaccines are available, significant attention has shifted to accelerating worldwide immunization strategies. The slow progression of such programs, along with the emergence of new SARS-CoV-2 variants that already show some degree of escape to the vaccine-induced immune response, highlights the relevance and urgency of identifying novel therapies [24–26]. Accordingly, current therapeutic approaches are insufficient to prevent extended periods of severe disease and progression to chronic lung injury and fibrosis in those who have recovered [27]. There is still an urgent need to improve supportive care in severe cases of COVID-19 and identify effective treatments that can prevent deterioration and decrease the mortality rate.

Cell-based therapies are currently being tested in clinical trials or on a compassionate-use basis to increase the survival of patients with severe COVID-19 pneumonia [28]. These therapies are based on the transfer of specific types of cells to control inflammation and stimulate endogenous repair or regenerative mechanisms. To date, most clinical protocols have evaluated the use of mesenchymal stromal cells (MSCs) obtained from different tissue sources, including the umbilical cord, adipose tissue, and bone marrow. In this review, we discuss the rationale, mechanisms of action, potential risks and benefits, and current challenges in translating MSC-based therapies into the armamentarium of therapeutic options against severe COVID-19.

## Pathophysiology of COVID-19

Current understanding of the pathophysiology of COVID-19 has been established through a comparative analysis of extensive data from previous studies on SARS-CoV and Middle East respiratory syndrome-CoV, along with reports of experimental and postmortem studies of SARS-CoV-2 infection. Viral particles enter target cells through an interaction of the coronavirus spike (S) protein with host cell angiotensin-converting enzyme 2 (ACE2), a proteolytic process that involves the transmembrane protease, serine 2 (TMPRSS2), followed by a cascade of intracellular signaling [29]. Although the respiratory tract has been shown to serve as an initial reservoir for viral infection and replication—especially the nasal and laryngeal mucosa—ACE2 receptors are located not only in lung structural cells but also in epithelial and endothelial cells of the heart, bowel, kidney, and brain, as well as vascular smooth muscle cells. Therefore, the clinical spectrum of COVID-19 is not limited to local pneumonia, but rather represents multiple organ involvement with potential for systemic complications [4, 30, 31].

Under normal physiological conditions, ACE2 has a protective role in lung tissue because it is a component of the renin-angiotensin-aldosterone system responsible for converting angiotensin II to angiotensin [1–7], which has vasodilator and antifibrotic activities [32, 33]. However, the interaction of SARS-CoV-2 with ACE2 prevents the latter from exerting its protective activity, resulting in dysfunction of the renin-angiotensin-aldosterone system and vasoconstrictive, pro-inflammatory, and pro-fibrotic actions due to the accumulation of angiotensin II [34]. Interestingly, ACE2 demethylation status seems to increase after SARS-CoV-2 infection, increasing ACE2 gene expression, possibly in response to exacerbated oxidative stress [35].

Several pathological mechanisms are involved in the disease progression and multiple organ dysfunction in COVID-19. These include direct viral toxicity, cell death mechanisms, endothelial dysfunction, thromboinflammation, dysregulation of the immune response, and tissue fibrosis [32]. Individuals who progress to acute respiratory failure associated with COVID-19 may develop an intense systemic inflammatory process in response to rapid viral replication and cell injury [36]. In lung tissue, postmortem studies have demonstrated diffuse alveolar damage, hyaline membrane formation, massive capillary congestion, microthrombi, inflammatory infiltrates, and consolidation with extensive fibrosis. In peripheral blood, lymphopenia is observed with substantially reduced CD4^+^ and CD8^+^ cell counts; however, these lymphocytes are activated, with an increase in the Th17 cell population [11, 37].

Cell death and inflammation appear to be associated with the pathogenesis of SARS-CoV-2 infection. SARS-CoV-2 infection has been shown to induce caspase-8 activation, a key regulator of different types of cell death, including apoptosis, pyroptosis, and necroptosis, and convert the pro-inflammatory cytokine IL-1β into its bioactive form. Moreover, the combination of increased production and release of combined TNF-α and IFN-γ has been linked with a process of PANptosis [38–40]. It has been shown that the SARS-CoV-2 accessory protein ORF-3 induces apoptosis through the extrinsic pathway, leading to caspase-8 cleavage without interfering with Bcl-2 levels [41]. Evidence of extensive apoptotic signals was detected in the lung tissues of humans and nonhuman primates following SARS-CoV-2 infection [42]. Pyroptosis is also induced in SARS-CoV-2-infected cells, triggered by inflammasome-mediated caspase 1/4/5/11 activation [43]. Caspase 1-dependent pyroptosis and the processing and release of the inflammatory cytokines IL-18 and IL-1β [44] induce interferon (IFN)-γ production and activation of T cells and macrophages, among others [45].

Type I IFN (IFN-α, IFN-β) activation is triggered by viral entry, but patients that progress to severe COVID-19 seem to present a delayed type I IFN response [46, 47]. While type I IFN products play important roles in early-phase antiviral defense, a delayed response has been associated with increased viral replication, intense parenchymal influx of inflammatory cells, and overexpression of pro-inflammatory cytokines [48].

Patients with severe COVID-19 show increased levels of IL-6, IL-8, IL-10, IL-2R, and tumor necrosis factor (TNF)-α compared to those with mild to moderate disease [49]. High concentrations of pro-inflammatory cytokines are associated with disease severity and T cell depletion. Lymphopenia, which affects both CD4 and CD8 subsets, is a very frequent finding. In severe/critical COVID-19 cases, T cell dysregulation has been shown, with the expression of hyperactivation markers and lower frequencies of terminally differentiated T cell subsets [50]. This contributes to a failure to adequate viral infection control that, combined with a hyperinflammatory state, contributes to more alveolar damage, establishment of ARDS, vascular hyperpermeability, and progression to multiorgan failure [51].

These findings suggest that the use of a therapeutic approach that suppresses production of pro-inflammatory cytokines and reduces the exuberant inflammatory response may be the key to reducing multiple organ dysfunction and increasing the survival of individuals with severe COVID-19. Nevertheless, this should be accompanied by an effective antiviral to avoid a superinfection state. Other crucial mechanistic targets should focus on vascular dysfunction, oxidative stress, and coagulation to promote repair mechanisms and restore pulmonary epithelial cell function.

## Mesenchymal stromal cells

MSCs were coined by Caplan in the 1990s to define mesenchymal stem cells, a type of multipotent stem cell with the potential to differentiate into cells of mesodermal origin, such as chondrocytes, osteocytes, and adipocytes and that could display pluripotency under certain in vitro conditions [52]. After the acknowledgment that MSCs are not clonal and are actually a heterogeneous population by nature, the acronym MSCs were then redefined to fit the currently accepted nomenclature: mesenchymal stromal cells. Finally, data collected from the application of MSCs in regenerative medicine have clearly shown that multipotency does not explain the beneficial actions exerted by these cells [53]. MSCs release anti-inflammatory, antibacterial, antiviral, and immunoregulatory factors locally at the lesion site [54]. Studies have demonstrated that, in response to tissue damage signals, MSCs release paracrine/endocrine factors that help control inflammation and stimulate tissue regeneration by endogenous mechanisms [55]. The recognition of these immunomodulatory/trophic actions with beneficial effects and migratory capacity in different tissues makes MSCs a unique therapeutic strategy [56, 57]. These MSC characteristics have also led their original describer, Caplan, to call for a further nomenclature change to medicinal signaling cells [53].

Nomenclature issues aside, the putative role of MSCs in vivo would be to participate in tissue homeostasis and to support tissue-resident stem cells (i.e., hematopoietic stem cells in the bone marrow), in addition to being a source of specialized cells of mesodermal lineage. The regulation of tissue homeostasis is mediated by cell signaling through the production of trophic factors, release of extracellular vesicles (EVs), etc. [58]. MSCs are morphologically similar to fibroblasts and strongly adhere to plastic surfaces [59]. MSCs also express some of their markers with other stromal cells, which makes them difficult to identify in vivo. However, some studies have suggested that pericytes are their in vivo counterparts [60].

MSCs can be obtained from different sources, including adult tissues (such as bone marrow and adipose tissue) and perinatal tissues (such as the umbilical cord) [61]. MSCs are characterized by immunophenotyping and functional assays, and multipotency was demonstrated by chondrogenic, osteogenic, and adipogenic differentiation assays in vitro. In 2006, a consensus statement by the International Society for Cellular Therapy established minimum criteria for identification of human MSCs based on the characteristics of these cells: (1) adhesion to plastic when maintained in standard culture conditions, fibroblast-like morphology; (2) expression (> 95%) of the surface molecules CD105, CD73, and CD90, but not of hematopoietic markers (< 2%), such as CD45, CD34, CD14 or CD11b, CD79 or CD19, and HLA-DR; and (3) ability to differentiate into osteoblasts, adipocytes, and chondroblasts in vitro [52]. This minimal characterization should be complemented by evaluation of additional cell markers and biological properties (e.g., potency assays) that may be relevant in the context of each intended clinical application.

**Fig. 1:**
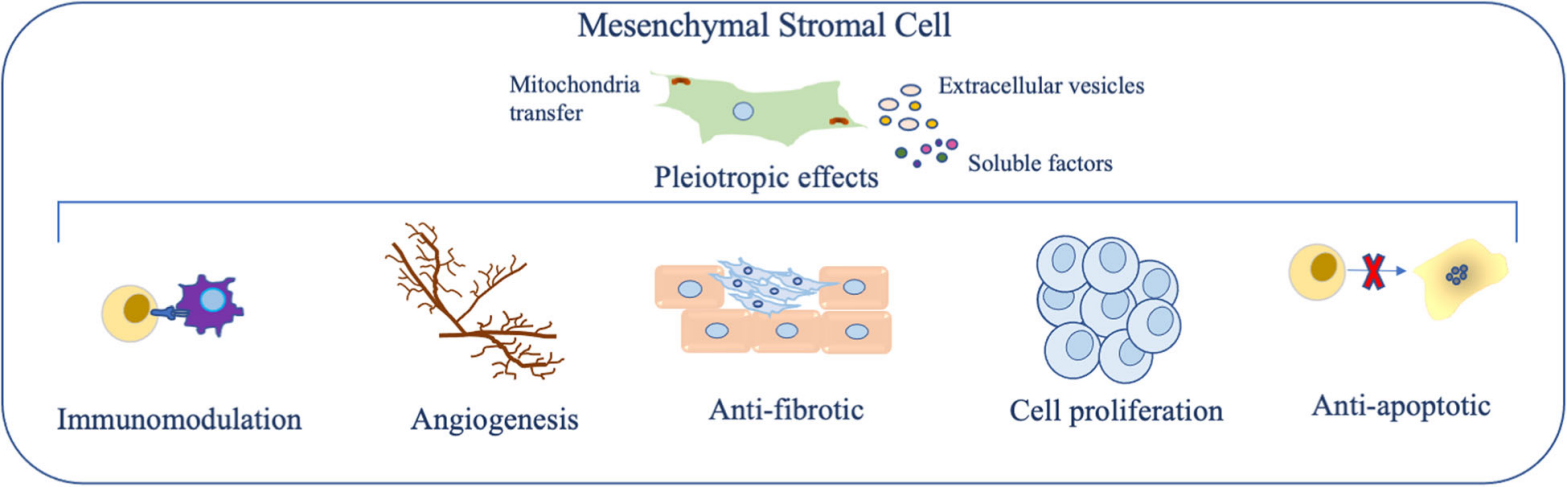
Mechanisms of action of mesenchymal stromal cells

**Fig. 2:**
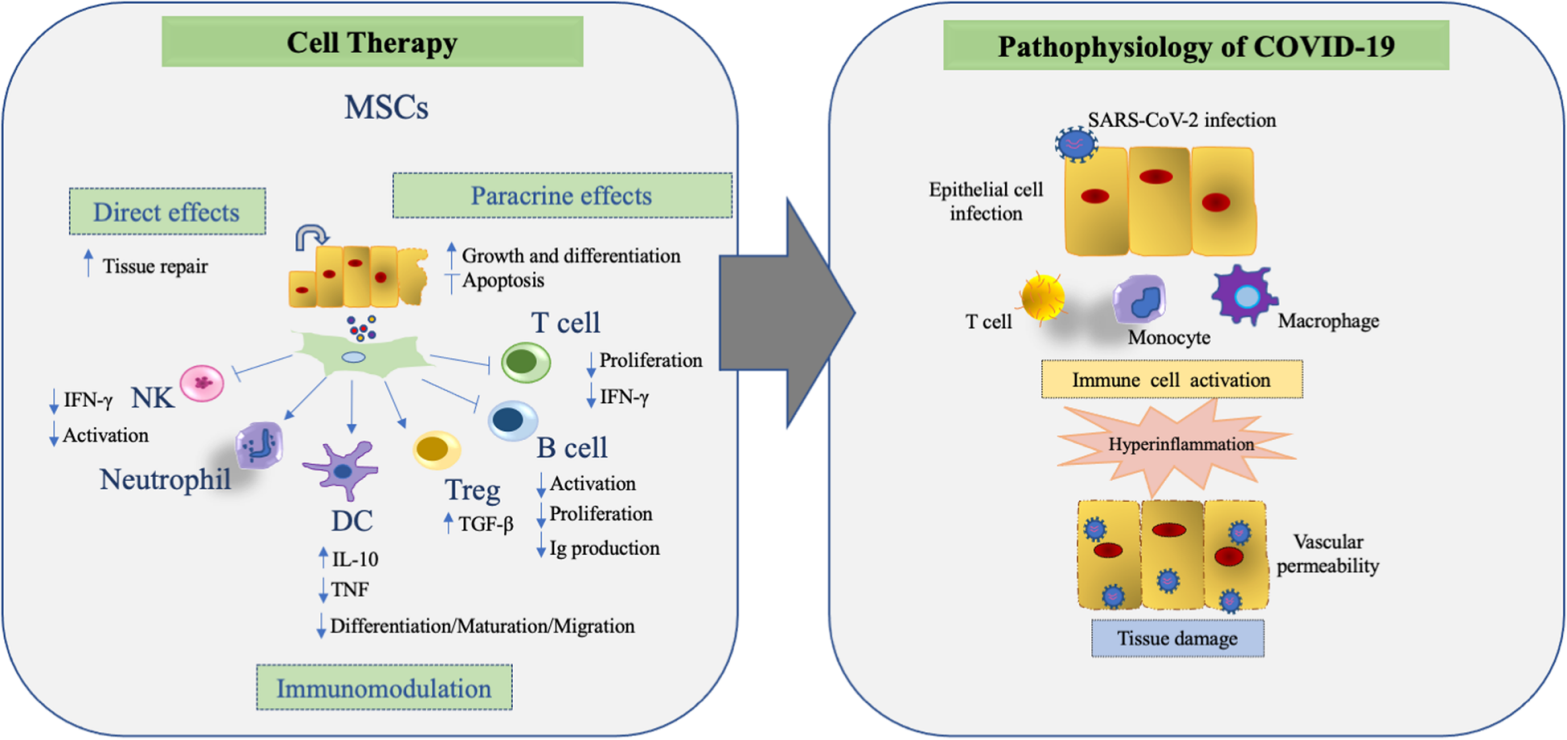
Mesenchymal stromal cells (MSCs) and immune modulation in COVID-19. DC, dendritic cell; IFN-γ, interferon-γ; IL, interleukin; TGF-β, transforming growth factor-β; TNF, tumor necrosis factor; Treg, regulatory T cell

The clinical applications of MSCs currently under investigation or authorized for marketing are based on their biological functions, including (1) high proliferative capacity, allowing sufficient in vitro expansion for utilization in autologous or allogeneic applications; (2) preferential migration toward inflammatory/injured sites, allowing both local and systemic administration, and (3) immunomodulatory/trophic actions [58] (Figs. 1 and 2).

## MSC-based therapy for COVID-19 pneumonia: potential mechanisms of action and therapeutic effects

In experimental ARDS, MSCs have been shown to promote anti-inflammatory and antimicrobial effects, tissue repair, and restoration of the alveolar epithelium and vascular endothelium via paracrine factors [62, 63]. Given that the mechanism of COVID-19 respiratory failure has certain similarities with ARDS, MSC therapy may improve lung function and promote endogenous repair in alveolar epithelial and vascular endothelial cells. The mechanisms by which MSCs exert these effects include paracrine activity, transfer of organelles and molecules (such as RNAs, microRNAs, proteins, peptides, cytokines, growth factors), and release of EVs [64, 65]. Depending on the environment they encounter, MSCs release mediators with antiapoptotic, immunomodulatory, antifibrotic, chemoattractant, or proangiogenic effects, among others. Interestingly, MSCs do not express ACE2 and are therefore relatively protected from SARS-CoV-2 infection [66].

The major mechanisms underlying MSC-based therapy rely on their interactions with target cells in injured tissues [67]. Thus, the route of administration is key to the outcome of cell therapies. Intravenous administration has been the most utilized, including in studies on COVID-19 and ARDS [68]. This is particularly important because individuals with severe COVID-19 are already extremely debilitated, and therefore, a peripheral vein could be easily accessed to infuse MSCs intravenously. Moreover, MSC biodistribution studies in animals have consistently shown four main results after intravenous administration of MSCs: (1) widespread systemic distribution of cells; (2) early accumulation in the lungs, followed by the liver and spleen; (3) directed migration toward injury sites; and (4) short-term persistence in tissues after infusion [69–74]. Interestingly, similar results were observed in humans [75]. The accumulation of intravenously administered MSCs in the lungs is the result of the initial capillary network that these cells first encounter and where they may become trapped, due not only to their size but also to the increased expression of adhesion molecules and chemokines (such as stromal cell–derived factor-1, a known chemotactic agent for MSCs) in the setting of injury and inflammation [76, 77].

Nevertheless, the brief persistence of MSCs in lung tissue might be explained by the fact that most infused cells actually become apoptotic and undergo phagocytosis by alveolar macrophages. Interestingly, this is another mechanism by which MSCs can shift inflammatory macrophages (M1) toward an anti-inflammatory or regulatory (M2) phenotype [78, 79]. As stated earlier, MSC-based therapy may contribute to a reduction in the levels of pro-inflammatory cytokines and chemokines in COVID-19 by responding to the hyperinflammatory microenvironment through the release of paracrine/immunomodulatory factors, including transforming growth factor-β, hepatocyte growth factor, indoleamine 2,3-dioxygenase, and IL-10 [36, 66]. Some studies have also reported the involvement of galectins, such as Gal-3, in MSC-mediated immunomodulation [80–82]. Indeed, these effects not only could be useful in treating COVID-19 pneumonia, but could also have a positive impact on the extrapulmonary complications of COVID-19. Indeed, MSCs present pro-survival actions, due to their ability to modulate processes of regulated cell death, including apoptosis, necroptosis, and pyroptosis, which are involved in the pathophysiology of COVID-19 [38, 40].

In addition to the abovementioned properties, MSCs release EVs (i.e., exosomes and microvesicles) to communicate with the surrounding microenvironment and exert pro-regenerative and immunomodulatory functions. The released EVs carry molecules such as cytokines, growth factors, chemokines, microRNAs, RNAs, enzymes, and hormones [83]. Interestingly, MSCs also act by transferring their healthy mitochondria to host cells through EVs, membrane nanotunneling, or gap junctions, a mechanism that can help restore normal epithelial and immune cell functions [84]. Transferred mitochondria are functionally active and dampen oxidative stress in recipient cells, restoring normal epithelial cell functions, such as surfactant secretion, and increasing phagocytic activity in macrophages [85]. Oxidative stress is associated with hyperinflammation in a reciprocal feedback loop, which has been shown to boost alveolar tissue damage in COVID-19 [86–90]. Data from studies on preclinical models of lung diseases, such as acute lung injury, suggest that mitochondrial transfer of MSCs occurs in vivo and may be in part responsible for certain beneficial effects observed in these models [91–93].

Besides immunomodulatory mechanisms, MSCs have demonstrated certain antiviral activity, as reviewed elsewhere [28]. In viral infections, MSCs stimulate the expression of IFN-I, which leads to an antiviral response in cells [94]. Besides the expression of IFN-stimulated genes (ISGs), MSCs also secrete indoleamine-2,3-dioxygenase (IDO) in response to pro-inflammatory microenvironments [95], which is one of the main immunomodulatory mechanisms of MSCs and has also been shown to possess antiviral properties [96]. Finally, miRNA delivery through the release of EVs may interfere with viral replication [97].

A recent preclinical trial in a sheep model of ARDS demonstrated a significant reduction in lung injury after treatment with MSCs [98]. MSCs were also able to attenuate the progression of chronic obstructive pulmonary disease (COPD), reducing endothelial cell apoptosis and oxidative stress in rats [99]. MSCs promote the transfer of mitochondria to cells that are damaged by oxidative stress, improving their mitochondrial bioenergetics, inducing cell regeneration, and improving the function of several organs, such as the lungs, heart, and brain [100]. In addition, Romieu-Mourez et al. demonstrated in vivo that toll-like receptor–activated MSCs increased immune responses and could be used in a cell-based vaccine [101]. Khatri et al. [102] found that systemic administration of MSC-EVs in a pig model of influenza virus–induced acute lung injury was able to significantly reduce the infiltration of inflammatory cells to the lungs and reduce the death of alveolar epithelium cells. They also found that MSC-EVs exhibited an immunomodulatory effect by suppressing TNF-α and increasing IL-10 secretion in the alveolus after injury.

Another MSC source relies on the differentiation of human-induced pluripotent stem cells (iPSCs). These iPSC-MSCs have been shown to suppress lung inflammation and decrease mitochondrial dysfunction and oxidative stress [92, 93], which are key factors in the pathophysiology of COPD [103] and also play an important role in infections with viruses such as influenza [104], human papillomavirus [105], SARS-CoV [106], and SARS-CoV-2 [107].

To date, there are no studies showing the effects of MSCs on inflammation or oxidative stress in SARS-CoV-2, whether in vivo or in vitro; however, the beneficial effects of MSCs on the inflammatory profile of other viral infections have been demonstrated. In this context, MSC administration reduced lung inflammation, increased body weight, and improved survival in H5N1 virus-infected aged mice [108] and was effective in restoring impaired alveolar fluid clearance and protein permeability of H5N1-infected human alveolar epithelial cells [109]. Similarly, MSCs suppressed pulmonary edema and inflammation and improved gas exchange in mice infected with the H9N2 avian subtype of influenza [110]. However, MSCs were not effective in reducing lung inflammation or increasing survival in influenza virus–infected mice [111] and had no beneficial effect on H1N1 infection [112]. Further investigations are needed to better understand the differences in the therapeutic efficacy of specific viruses.

## Potential risks of MSC-based therapies

Despite extensive investigations of cell-based therapies, MSC administration is not completely free of potential risks. Adverse events might include infusion reactions, allergic reactions, secondary infections, and thromboembolic events [113]. The latter is a particular concern in the context of COVID-19 because the disease has been associated with a hypercoagulable state [114]. These risks are increased when considering intravenous injections and can be reduced by utilizing other administration routes, such as intratracheal administration. The risk of environmental contamination with aerosols, along with the poor feasibility of administering fluids to hypoxemic patients, however, has favored the use of the intravenous route of administration in most ongoing studies on cell-based therapy for COVID-19. The safety intravenous route for MSC administration was previously demonstrated in ARDS, even at high cell doses [115].

The potential risk of thromboembolic events associated with intravenous infusions of high doses of MSCs has long been known in animal studies, with a few reports in humans [116, 117]. This procoagulant activity is related to the high expression of tissue factor (TF/CD142), which can be found in some MSC isolates and sources and leads to an increased risk of instant blood-mediated inflammatory reaction and clot formation [118]. Such events are also known to occur after intravenous infusion of pancreatic islets or hepatocytes [119, 120]. Among MSCs, adipose tissue– and amniotic fluid–derived MSCs consistently show high expression of TF, whereas negligible expression of TF is found in MSCs from other sources, such as the bone marrow [121]. Considering that the expression level of TF is highly variable among MSC isolates and tends to increase with passage, duration of culture expansion, and poor quality of culture conditions, it is reasonable that MSCs intended for clinical use be checked and evaluated for TF expression and undergo ex vivo coagulation studies (e.g., thromboelastography) [118, 122].

MSC-based products offer several advantages for clinical application, including the ability to be cryopreserved, isolated from allogeneic donors, and available for off-the-shelf use to treat COVID-19. Allogeneic infusion of MSCs from third parties is allowed because these cells do not express ABO antigens, are immune-evasive, and show low immunogenicity due to the very low expression of major histocompatibility complex [123]. Repeated administration of MSCs, however, leads to the detection of anti-HLA antibodies, suggesting potential alloreactivity [124]. Although this finding might compromise the long-term permanence of MSCs and their progeny in regenerative therapies in which cell replacement is required, this would not compromise therapeutic efficacy in acute disease settings, such as COVID-19, in which immunomodulatory and pro-regenerative actions are dependent on short-term cell signaling [125]. Finally, it is important to emphasize that although MSCs do not express ABO antigens, these cells can be “contaminated” with ABO antigens in certain bioprocessing protocols that utilize human serum as a supplement. Alternatives, such as the use of platelet lysate [126], are available to establish an MSC bioprocess free of such contaminants.

Cryopreserved MSC products have great advantages over fresh products in terms of their feasibility for clinical applications and off-the-shelf availability, because they are fully characterized and confirmed sterile before infusion, characteristics that are simply not attainable with fresh products [127]. They are also amenable to transport between production sites and infusion sites without losing viability, as long as the temperature is monitored. Typically, these products are transported and thawed at the bedside, allowing infusion to begin within minutes to maintain cell viability. However, this procedure requires cell infusion in a cryoprotectant solution, which commonly contains dimethyl sulfoxide. This may lead to an acute toxicity syndrome that includes skin reactions, headache, dizziness, nausea, vomiting, and allergic reactions [128]. Premedication with antihistamines, commonly used in the transplantation of hematopoietic progenitor cells in clinical practice, can prevent these events [129].

The risk of secondary infections is potentiated by the use of fresh cell-based products when the results of microbiological testing are released only after the product has been infused into a patient. There is also the theoretical possibility that a state of immunosuppression induced by MSCs would allow pathogens to evade the immune response and establish secondary infections, especially in critically ill patients [130, 131]. Data from robust clinical trials of MSC-based therapies in COVID-19 have yet to be published; therefore, this possibility is speculative and conflicts with data from several preclinical studies in which MSC treatment has been shown to help the immune system fight bacteria through direct and indirect antimicrobial actions [132].

Indeed, cell-based therapies have been associated with a risk of long-term persistence, ectopic tissue formation, or tumorigenesis. Although this risk is much more strongly linked with pluripotent cell–derived products, ex vivo expansion may be associated with genetic instability and changes in cell behavior [133]. This highlights the importance of using cells that have not undergone extensive ex vivo population doublings and monitoring the genetic stability of MSCs, at least by karyotyping [134]. MSCs have been extensively used in clinical protocols, and thousands of patients have received such therapies, which are considered quite safe in long-term analyses as long as all controls have been followed during bioprocessing [135].

Zheng et al. [136] randomized 12 patients with ARDS 1:1 to receive allogeneic adipose–derived MSCs or placebo. Patients received a single intravenous dose of 1 × 10^6^ cells/kg body weight or saline. In relation to MSC administration, there were no infusion toxicities or serious adverse events and no significant differences in the overall number of adverse events between the groups. Duration of hospital stay, ventilator-free days, and ICU-free days at day 28 after treatment were similar. There were no changes in the biomarkers of interest in the placebo group. In the MSC group, serum surfactant protein D (SP-D) levels at day 5 were significantly lower than those on day 0 (*P* = 0.027), although the changes in IL-8 levels were not significant. The IL-6 levels at day 5 showed a nonsignificant tendency toward lower levels compared with baseline. The study reported a good safety profile of adipose-derived MSC administration in patients with ARDS.

In 2015, Wilson et al. conducted a multicenter, open-label, dose-escalation, phase 1 clinical trial in patients with ARDS [137]. The first three patients were treated with low-dose MSCs (1 × 10^6^ cells/kg predicted body weight [PBW]), the next three patients received intermediate-dose MSCs (5 × 10^6^ cells/kg PBW), and the final three patients received high-dose MSCs (10 × 10^6^ cells/kg PBW). Pre-specified infusion-associated events or treatment-related adverse events were not reported in any of the nine patients. However, subsequent identification of serious adverse events occurred in three patients during the weeks after the infusion: one of them died on study day 9, another died on study day 31, and one patient was revealed to have multiple embolic age-indeterminate infarcts of the spleen, kidneys, and brain that were thought to have occurred before the MSC infusion based on magnetic resonance imaging results. None of these severe adverse events were considered MSC-related.

Matthay et al. performed a phase 2a randomized clinical trial [115]. Sixty patients were randomly assigned 2:1 to receive either 10 × 10^6^/kg PBW MSCs or placebo. None of the patients experienced any of the predefined MSC-related hemodynamic or respiratory adverse events. One patient in the MSC group died within 24 h of MSC infusion, which was judged to be unrelated. Furthermore, 28-day mortality did not differ between the groups (30% in the MSC group versus 15% in the placebo group; odds ratio, 2.4; 95% confidence interval, 0.5–15.1).

To study influenza-associated ARDS, Chen et al. [138] enrolled 61 patients with H7N9-induced ARDS in a nonrandomized clinical trial; 17 patients received allogeneic menstrual blood–derived MSCs. The experimental group had lower mortality rates (17.6% versus 54.5% in the experimental and control groups, respectively). These data indicate that MSC therapy is a safe and effective treatment for patients with severe lung disease induced by H7N9. In addition, MSC transplantation did not result in harmful effects over 5 years of follow-up.

Finally, a recent meta-analysis of MSC-based therapies to treat ARDS revealed a favorable trend toward reduced mortality and inflammatory biomarkers, as well as improved pulmonary function and radiographic data. Moreover, despite significant variation in terms of protocols, cell sources, and dosage, no serious adverse events were reported in the studies evaluated, which included a total of 117 patients [68].

## Evidence from clinical studies

### COVID-19

Clinical experience with cell-based therapies for COVID-19 remains limited. Although multiple clinical trials are registered in public databases, such as clinicaltrials.gov (Table 1), only preliminary results from a few clinical studies have been published (Table 2) [66, 130, 139–154]. Many translational gaps have led to a tremendous variety in protocols, cell sources, and dosage regimens; there is also great heterogeneity in clinical presentations and stages of the disease. For instance, current knowledge is not sufficient to give support to any of the following aspects: (1) cell dose (which can be fixed or calculated by body mass); (2) use of cryopreserved versus fresh cells (i.e., whether MSCs can recover and function in vivo immediately after being thawed and infused); (3) whether any specific cell source might be more suitable, safe, and effective; and (4) the therapeutic window and expected results [28, 155].

**Table 1:** Clinical trials of cell-based therapies in COVID-19 patients registered in clinicaltrials.gov

	Title	ID	Interventions	Primary outcome	Age	Phases	Enrollment	Location
1	The Mesenchymal coviD-19 Trial: a Pilot Study to Investigate Early Efficacy of MSCs in Adults With COVID-19 (MEND	NCT04537351	CYP-001	Trend in trajectory of PaO_2_/FiO_2_ ratio (P/F ratio) between in 7 days	< 18 years	Phase I Phase II	24	Australia
2	Treatment of Covid-19 Associated Pneumonia with Allogenic Pooled Olfactory Mucosa-derived Mesenchymal Stem Cells	NCT04382547	Allogenic pooled olfactory mucosa-derived MSCs *versus* control	Treatment: Number of cured patients in 3 weeks	18 to 70 Years	Phase I Phase II	40	Belarus
3	Mesenchymal Stromal Cell Therapy for Severe Covid-19 Infection	NCT04445454	BM-MSC	Adverse events in 28 days	18 to 70 years	Phase 1 Phase 2	20	Belgium
4	MSC-based Therapy in COVID-19-associated Acute Respiratory Distress Syndrome	NCT04525378	MSCs	Intrahospital mortality, incidence of adverse events in 28 days and quantification of biomarkers	≥ 18 years	Phase 1	20	Brazil
5	This is phase II study to assess the efficacy of NestaCell® (mesenchymal stem cell) to treat severe COVID-19 pneumonia	NCT04315987	NestaCell® (MSCs) *versus* Placebo	Change in clinical condition in 10 days	≥ 18 years	Phase II	90	Brazil
6	Safety and Feasibility of Allogenic MSC in the Treatment of COVID-19	NCT04467047	MSCs	Overall survival in 60 days	Child, Adult, Older Adult	Phase I	10	Brazil
7	Cellular Immuno-Therapy for COVID-19 Acute Respiratory Distress Syndrome - Vanguard (CIRCA-19)	NCT04400032	BMCs	Number of Participants with Treatment-Related Adverse Events as Assessed by CTCAE v4.0 to determine the maximum feasible tolerated dose (MFTD) of BM-MSCs given to patients with COVID-19	≥ 18 years	Phase I	9	Canada
8	Safety and Efficacy of Mesenchymal Stem Cells in the Management of Severe COVID-19 Pneumonia	NCT04429763	UC-MSCs *versus* Placebo	Clinical deterioration or death in 4 weeks	18 to 79 Years	Phase II	30	Colombia
9	Safety and Efficacy of Intravenous Wharton's Jelly Derived Mesenchymal Stem Cells in Acute Respiratory Distress Syndrome Due to COVID 19	NCT04390152	WJ-MSC *versus* control	Evaluation of efficacy of WJ-MSC defined by mortality at 28 days of application	18 to 80 years	Phase I Phase II	40	Colombia
10	Mesenchymal Stem Cell Treatment for Pneumonia Patients Infected With COVID-19	NCT04252118	MSCs	Size of lesion area by chest radiograph or CT and side effects in 28 days	18 to 70 years	Phase 1	20	China
11	Clinical Research of Human Mesenchymal Stem Cells in the Treatment of COVID-19 Pneumonia	NCT04339660	UC-MSCs *versus* Placebo	Immune function and blood oxygen saturation	18 to 75 years	Phase 1 Phase 2	30	China
12	Safety and Effectiveness of Mesenchymal Stem Cells in the Treatment of Pneumonia of Coronavirus Disease 2019	NCT04371601	MSCs versus control	Improvement of pulmonary function in 12 months	18 to 70 years	Phase I	60	China
13	Safety and Efficacy Study of Allogeneic Human Dental Pulp Mesenchymal Stem Cells to Treat Severe COVID-19 Patients	NCT04336254	DPSCs *versus* Placebo	Clinical improvement in 28 days	18 to 65 years	Phase 1 Phase 2	20	China
14	Study of Human Umbilical Cord Mesenchymal Stem Cells in the Treatment of Severe COVID-19	NCT04273646	UC-MSCs *versus* Placebo	Evaluation of pneumonia improvement	18 to 65 years	Not Applicable	48	China
15	Bone Marrow-Derived Mesenchymal Stem Cell Treatment for Severe Patients with Coronavirus Disease 2019 (COVID-19)	NCT04346368	BM-MSCs *versus* Placebo	Changes of oxygenation index (PaO_2_/FiO_2_) at baseline, 6-hour, day 1, day 3 week 1, week 2, week 4, month 6 Evaluation of pneumonia improvement	18 to 75 years	Phase I Phase II	20	China
16	Pilot Clinical Study on Inhalation of Mesenchymal Stem Cells Exosomes Treating Severe Novel Coronavirus Pneumonia	NCT04276987	MSCs-Exo	Safety evaluation within 28 days after first treatment, including frequency of adverse reaction (AE) and severe adverse reaction (SAE)	18 to 75 years	Phase I	24	China
15	Novel Coronavirus Induced Severe Pneumonia Treated by Dental Pulp Mesenchymal Stem Cells	NCT04302519	Dental pulp MSCs	Disappear time of ground-glass shadow in the lungs in 14 days	18 to 75 years	Phase I	24	China
16	Umbilical Cord (UC)-Derived Mesenchymal Stem Cells (MSCs) Treatment for the 2019-novel Coronavirus (nCOV) Pneumonia	NCT04269525	UC-MSCs	Oxygenation index on after 14 days	18 to 80 years	Phase II	16	China
17	Treatment with Human Umbilical Cord-derived Mesenchymal Stem Cells for Severe Corona Virus Disease 2019 (COVID-19)	NCT04288102	UC-MSCs *versus* Placebo	Change in lesion proportion (%) of full lung volume from baseline to day 28 Evaluation of pneumonia improvement	18 to 75 years	Phase II	100	China
18	Cell Therapy Using Umbilical Cord-derived Mesenchymal Stromal Cells in SARS-CoV-2-related ARDS	NCT04333368	UC-MSC *versus* NaCl 0.9%	PaO_2_/FiO_2_ ratio from baseline to day 7	≥ 18 years	Phase 1 Phase 2	40	France
19	Mesenchymal Stem Cells (MSCs) in Inflammation-Resolution Programs of Coronavirus Disease 2019 (COVID-19) Induced Acute Respiratory Distress Syndrome (ARDS)	NCT04377334	BM-MSCs *versus* control	Lung injury score in 10 days Improvement of lung injury score (LIS), 0-16 points, severity increasing with higher points	≥ 18 years	Phase II	40	Germany
20	Administration of Allogenic UC-MSCs as Adjuvant Therapy for Critically-Ill COVID-19 Patients	NCT04457609	UC-MSCs *versus* control group	Clinical improvement in 15 days	18 to 95 years	Phase I	40	Indonesia
21	Treatment of Severe COVID-19 Patients using Secretome of Hypoxia-Mesenchymal Stem Cells in Indonesia	NCT04753476	S-MSCs *versus* control	Change in patients clinical manifestation in 1 months (mild, moderate, or severe)	Child, Adult, Older Adult	Phase II	48	Indonesia
22	Therapeutic Study to Evaluate the Safety and Efficacy of DW-MSC in COVID-19 Patients (DW-MSC)	NCT04535856	MSCs *versus* Placebo	Treatment-emergent adverse event All adverse reactions in treatment group in 28 days	≥ 19 years	Phase I	9	Indonesia
23	Mesenchymal Stem Cell Therapy for SARS-CoV-2-related ARDS	NCT04366063	MSCs and EVs	Adverse events in 28 days and blood oxygen saturation	18 to 65 years	Phase 2 Phase 3	60	Iran
24	An Exploratory Study of ADR-001 in Patients with Severe Pneumonia Caused by SARS-CoV-2 Infection (COVID-19)	NCT04522986	MSCs	Safety: adverse event in 12 weeks	≥ 20 years	Phase I	6	Japan
25	Mesenchymal Stem Cell for Acute Respiratory Distress Syndrome Due for COVID-19	NCT04416139	UC-MSCs	PaO_2_/FiO_2_ ratio and clinical changes in 3 weeks	18 years and older	Phase 2	10	Mexico
26								
27	Mesenchymal Stem Cells in Patients Diagnosed With COVID-19	NCT04611256	MSCs *versus* control	Change form baseline in Arterial oxygen saturation up to 25 days Pulmonary lesion area will be taken by a chest X-ray or computed axial tomography	18 to 65 years	Phase I	20	Mexico
28	Use of Mesenchymal Stem Cells in Acute Respiratory Distress Syndrome Caused by COVID-19	NCT04456361	WJ-MSCs	Oxygen saturation Baseline, and at days 2, 4 and 14 post-treatment Number of patients with changes in percentage of resting oxygen saturation (%O2)	≥ 18 years	Phase I	9	Mexico
29	Efficacy of Intravenous Infusions of Stem Cells in the Treatment of COVID-19 Patients	NCT04437823	UC-MSCs	Adverse events, improvements in chest radiograph or chest CT scan in 30 days	30 to 70 years	Phase 2	20	Pakistan
30	Mesenchymal Stem Cell Infusion for COVID-19 Infection	NCT04444271	MSC *versus* Placebo	Overall survival in 30 days	10 years and older	Phase 2	20	Pakistan
31	Investigational Treatments for COVID-19 in Tertiary Care Hospital of Pakistan	NCT04492501	MSCs *versus* control	Survival in 28 days	18 to 90 years	Not Applicable	600	Pakistan
32	Treatment of Severe COVID-19 Pneumonia with Allogeneic Mesenchymal Stromal Cells (COVID_MSV)	NCT04361942	MSC *versus* Placebo	Proportion of patients who have achieved withdrawal of invasive mechanical ventilation in 7 days and mortality rate in 28 days	≥18 years	Phase 2	24	Spain
33	Clinical Trial of Allogeneic Mesenchymal Cells from Umbilical Cord Tissue in Patients With COVID-19	NCT04366271	UC-MSC *versus* Standard of care	Mortality rate in 28 days	40 to 80 years	Phase 2	106	Spain
34	Clinical Trial to Assess the Safety and Efficacy of Intravenous Administration of Allogeneic Adult Mesenchymal Stem Cells of Expanded Adipose Tissue in Patients With Severe Pneumonia Due to COVID-19	NCT04366323	AD-MSCs	Adverse event rate in 12 months; survival rate in 28 days	18 to 80 years	Phase 1 Phase 2	26	Spain
35	BAttLe Against COVID-19 Using MesenchYmal Stromal Cells	NCT04348461	AD-MSCs *versus* control	Efficacy of the administration of allogeneic AD- MSCs by survival rate in 28 days Safety of the administration of allogeneic AD-MSCs by adverse event rate in 6 months	≥ 18 years	Phase II	100	Spain
36	Efficacy and Safety Evaluation of Mesenchymal Stem Cells for the Treatment of Patients with Respiratory Distress Due to COVID-19 (COVIDMES)	NCT04390139	WJ-MSC *versus* Placebo	All-cause mortality at day 28	18 to 70 years	Phase I Phase II	30	Spain
37	Treatment of Severe COVID-19 Pneumonia with Allogeneic Mesenchymal Stromal Cells (COVID-MSV)	NCT04361942	MSCs *versus* Placebo	Proportion of patients who have achieved withdrawal of invasive mechanical ventilation in 7 days	≥ 18 years	Phase II	24	Spain
38	Mesenchymal Stromal Cell Therapy for The Treatment of Acute Respiratory Distress Syndrome	NCT04447833	BM-MSC	Incidence of pre-specified adverse events in 10 days	18 to 65 years	Phase 1	9	Sweden
39	Investigational Treatments for COVID-19 in Tertiary Care Hospital of Pakistan	NCT04492501	MSCs *versus* control	Survival in 28 days	18 to 90 years		600	Pakistan
40	Evaluation of Safety and Efficiency of Method of Exosome Inhalation in SARS-CoV-2 Associated Pneumonia	NCT04491240	EXO 1, EXO 2 *versus* Placebo	Number of participants with non-serious and serious adverse events during trial 30 days after clinic discharge Number of participants with non-serious and serious adverse during inhalation procedure after each inhalation during 10 days	18 to 65 years	Phase I Phase II	30	Russian
41	Safety and Efficiency of Method of Exosome Inhalation in COVID-19 Associated Pneumonia	NCT04602442	EXO 1, EXO 2 *versus* Placebo	Number of participants with non-serious and serious adverse events during trial in 2 months Number of participants with non-serious and serious adverse during inhalation procedure in 10 days during inhalation procedures	18 to 65 years	Phase II	90	Russian
42	Clinical Use of Stem Cells for the Treatment of Covid-19	NCT04392778	MSC *versus* control	Clinical improvement in 3 months	40 to 60 years	Phase 1 Phase 2	30	Turkey
43	Mesenchymal Stem Cells Therapy in Patients With COVID-19 Pneumonia	NCT04713878	MSCs	Clinical symptoms as respiratory distress	18 to 90 years	Not Applicable	21	Turkey
44	Repair of Acute Respiratory Distress Syndrome by Stromal Cell Administration (REALIST) (COVID-19)	NCT03042143	UC-MSCs *versus* Plasma-Lyte	Oxygenation index in 7 days and incidence of serious adverse events in 28 days	≥16 years	Phase 1 Phase 2	75	United Kingdom
45	Treatment of Coronavirus COVID-19 Pneumonia (Pathogen SARS-CoV-2) with Cryopreserved Allogeneic P_MMSCs and UC-MMSCs	NCT04461925	P-MMSCs *versus* Antibiotics/ Hormones/ Anticoagulant Therapy	PaO_2_/FiO_2_ ratio and mortality rate in 28 days	18 to 75 years	Phase 1 Phase 2	30	Ukraine
46	Use of UC-MSCs for COVID-19 Patients	NCT04355728	UC-MSCs + Heparin alongside best supportive care *versus* Vehicle + Heparin alongside best supportive care	Incidence of pre-specified infusion-associated adverse events in 5 days and incidence of severe adverse events in 90 days	≥18 years	Phase 1 Phase 2	24	United States
47	hCT-MSCs for COVID-19 ARDS	NCT04399889	UC-MSCs *versus* Standard care	Safety of the investigational product in 28 days	≥18 years	Phase 1 Phase 2	30	United States
48	Umbilical Cord Lining Stem Cells (ULSC) in Patients With COVID-19 ARDS (ULSC)	NCT04494386	UC-MSCs *versus* Placebo	Incidence of dose-limiting toxicity and adverse events in 12 months	≥18 years	Phase 1 Phase 2	60	United States
49	Cord Blood-Derived Mesenchymal Stem Cells for the Treatment of COVID-19 Related Acute Respiratory Distress Syndrome	NCT04565665	CB-MSC	Incidence of serious adverse events	≥18 years	Phase II	70	United States
50	Study of Intravenous Administration of Allogeneic Adipose-Derived Mesenchymal Stem Cells for COVID-19-Induced Acute Respiratory Distress	NCT04728698	COVI-MSCs *versus* Placebo	Mortality at day 28	≥ 18 years	Phase II	100	United States
51	A Randomized, Double-Blind, Placebo-Controlled Clinical Trial to Determine the Safety and Efficacy of Hope Biosciences Allogeneic Mesenchymal Stem Cell Therapy (HB-adMSCs) to Provide Protection Against COVID-19	NCT04348435	HB-adMSCs *versus* Placebos	Incidence of hospitalization for COVID-19 (week 0 through week 26 end of study Number of subjects that must be hospitalized for COVID-19 during the conduct of this study	≥ 18 years	Phase II	100	United States
52	Regenerative Medicine for COVID-19 and Flu-Elicited ARDS Using Longeveron Mesenchymal Stem Cells (LMSCs)	NCT04629105	LMSCs *versus* Placebo	Incidence of treatment-emergent serious adverse events (TE-SAEs) within 4 weeks after treatment	≥ 18 years	Phase I	70	United States
53	A Clinical Trial to Determine the Safety and Efficacy of Hope Biosciences Autologous Mesenchymal Stem Cell Therapy (HB-adMSCs) to Provide Protection Against COVID-19	NCT04349631	HB-adMSCs	Number of subjects that require hospitalization for COVID-19	≥ 65 years	Phase II	56	United States
54	Efficacy and Safety Study of Allogeneic HB-adMSCs for the Treatment of COVID-19	NCT04362189	HB-adMSC *versus* placebo	Interleukin-6, C-reactive protein, oxygenation, NF alpha, IL-10 from change from baseline and time to return to room air	≥ 18 years	Phase II	100	United States
55	Umbilical Cord Tissue (UC) Derived Mesenchymal Stem Cells (MSCs) Versus Placebo to Treat Acute Pulmonary Inflammation Due to COVID-19	NCT04490486	UC-MSCs *versus* Placebo	Safety of UCMSCs will be reported as the percentage of participants in each treatment group that experienced a treatment-related serious adverse events (SAEs)	≥ 18 years	Phase I	21	United States
56	A Phase II Study in Patients with Moderate to Severe ARDS Due to COVID-19	NCT04780685	hMSCs *versus* placebo	Survival in 14 days post-treatment	≥ 18 years	Phase II	40	United States
57	ACT-20 in Patients with Severe COVID-19 Pneumonia	NCT04398303	CT-20-MSC, or ACT-20-CM *versus* Placebo	Mortality day 30 post-treatment	18 to 85 years	Phase I Phase II	70	United States
58	MSCs in COVID-19 ARDS	NCT04371393	Remestemcel-L *versus* Placebo	Number of all-cause mortality within 30 days of randomization Change in IL6, IL-8, TNF-alpha inflammatory marker level in 7,14, 21, 30 days	18 to 75 years	Phase III	223	United States
59	Multiple Dosing of Mesenchymal Stromal Cells in Patients with ARDS (COVID-19)	NCT04466098	MSCs *versus* Placebo	Incidence of grade 3–5 infusional toxicities and predefined hemodynamic or respiratory adverse events related to the infusion of MSC in 6 hours of the start of the infusion	18 to 80 years	Phase II	30	United States
60	A Study of Cell Therapy in COVID-19 Subjects with Acute Kidney Injury Who Are Receiving Renal Replacement Therapy	NCT04445220	SBI-101 *versus* control	Safety and tolerability as measured by incidence of IP-related serious adverse events in 180	≥ 18 years	Phase I Phase II	22	United States
61	Study of Descartes-30 in Acute Respiratory Distress Syndrome	NCT04524962	Descartes 30	To assess the safety of Descartes-30 in patients with moderate-to-severe ARDS in 2 years	≥ 18 years	Phase I Phase II	30	United States
62	Mesenchymal Stem Cells for the Treatment of COVID-19	NCT04573270	Primepro *versus* Placebo	Survival rate in COVID-19 infected patients admitted to hospital for complications Contraction rate of COVID-19 in healthy healthcare workers following patients admitted to hospital for complications due to COVID-19	≥ 18 years	Phase I	40	United States
63	Mesenchymal Stromal Cells for the Treatment of SARS-CoV-2 Induced Acute Respiratory Failure (COVID-19 Disease)	NCT04362189	MSCs versus control	Treatment-related serious adverse events (tSAEs) 28 days post cell infusion and Change in clinical status in 14 days post infusion	≥ 18 years	Phase I Phase II	30	United State
64	Study of the Safety of Therapeutic Tx With Immunomodulatory MSC in Adults With COVID-19 Infection Requiring Mechanical Ventilation	NCT04397796	BM-Allo.MSC versus Placebo	Incidence of AEs, mortality, death, within 30 days of randomization Number of ventilator-free days in 60 days	≥ 18 years	Phase I	45	United States
65	The Use of Exosomes for the Treatment of Acute Respiratory Distress Syndrome or Novel Coronavirus Pneumonia Caused by COVID-19 (ARDOXSO)	NCT04798716	MSC-Exo	Evaluate the efficacy of ARDOXSO™ as an interventional exosome therapy in COVID-19 patients in 90 days	≥ 18 years	Phase I Phase II	55	United States
66	Adipose Mesenchymal Cells for Abatement of SARS-CoV-2 Respiratory Compromise in COVID-19 Disease	NCT04352803	AD-MSCs	Safety Incidence of unexpected adverse events within 28 days following IV administration of MSCs and efficacy	18 to 90 Years	Phase I	20	United States
67	Use of hUC-MSC Product (BX-U001) for the Treatment of COVID-19 With ARDS	NCT04452097	HU-MSCs *versus* Placebo	Incidence of infusion-related adverse events in day 3 Incidence of any treatment-emergent adverse events (TEAEs) and treatment-emergent serious adverse events (TE-SAEs) in day 28	18 to 80 years	Phase I Phase II	39	United States

*MSCs*, mesenchymal stem/stromal cells; *BM-MSCs*, bone marrow-derived mesenchymal stem cells; *UC-MSCs*, umbilical cord-derived mesenchymal stem cells; *ESCs*, embryonic stem cells; *WJ-MSCs*, Wharton’s jelly-derived mesenchymal stem cells; *P-MMSCs*, placenta-derived multipotent mesenchymal stem cells; *AD-MSCs*, adipose tissue-derived mesenchymal stem cells; *DPSCs*, dental pulp stem cells; *EVs-CDCs*, extracellular vesicles from cardiosphere-derived cells; *DSC*, decidual stroma cells; *MSCs-Exo*, mesenchymal stem cell derived exosomes; *hCT-MSCs*, human cord tissue mesenchymal stromal cells; *ULSC*, umbilical cord lining stem cells; *ARDS*, acute respiratory distress syndrome; *CB-MCS*, cord blood–derived mesenchymal stem cell; *S-MSCs*, secretome-MSCs; *COVI-MSC*, allogeneic culture-expanded adipose-derived mesenchymal stem cells (MSCs); *HB-adMSCs*, Hope Biosciences allogeneic adipose-derived mesenchymal stem cells; *LMSCs*, Longeveron mesenchymal stem cells; *HB-adMSCs*, autologous adipose-derived mesenchymal stem cells; *ACT-20-MSC*, allogenic human umbilical derived mesenchymal stem cells; *ACT-20-CM*, allogenic human umbilical derived mesenchymal stem cells in conditioned media; *CYP-001*, Cymerus mesenchymal stem cells (MSCs). Remestemcel-L is a third-party of adult human mesenchymal stem cells; SBI-101 is a combination product: allogeneic human mesenchymal stromal cells (MSCs) and an FDA-approved plasmapheresis device; Descartes 30, mesenchymal stem cells or MSCs RNA-engineered to secrete a combination of DNases. Primepro, umbilical cord–derived stem cell

**Table 2:** Reports of clinical use of MSCs in COVID-19 patients

Study phase/type	Country	Sample size	Patient characteristics	Cell product	Dose and administration regimen	Main findings	Adverse events	References
Pilot study	China	10 patients, *n =* 7 included in the cell therapy arm	COVID-19 pneumonia confirmed by RT-PCR, with no improvement under standard treatment	Allogeneic, UC-MSC	Single dose of 1 × 10^6^ MSCs/kg, i.v. infusion	Improvement of pulmonary function and symptoms, increase of peripheral lymphocytes, decrease in C-reactive protein, disappearance of overactivated cytokine-secreting immune cells, decrease of TNF-α levels and increase of IL-10 levels	No treatment-related adverse events	Leng et al, 2020 [66]
Phase 1, controlled, open label	China	41 patients, *n =* 12 included in the cell therapy arm	Severe COVID-19, clinical symptoms were not alleviated under standard treatment for 7 to 10 days	Allogeneic, UC-MSC	Single dose of 2 × 10^6^ MSCs/kg, i.v. infusion	Relief of clinical symptoms, reduction of inflammatory factors, increase of lymphocytes, patients with diabetes used less exogenous insulin after hUC-MSC infusion than usual	No treatment-related adverse events	Shu et al, 2020 [139]
Primary safety trial, open-label cohort study	USA	27 patients	Severe or critical COVID-19 SpO_2_ < 94% on room air (RA), with fever and dyspnea (*n =* 2); patients with SpO_2_ < 90% on RA or patients who required supplemental oxygen to maintain SpO_2_ < 94% (*n =* 21), and patients with hypoxic respiratory failure on mechanical ventilation (*n =* 4)	Allogeneic, MSC-Exo	15 ml of ExoFlo was added to 100 mL of normal saline, i.v. infusion Quantification and characterization of exosomes were not provided	Reversal of hypoxia, immune reconstitution, modulation of cytokine storm	No treatment-related adverse events	Sengupta et al, 2020 [140]
Case series/Retrospective study	China	25 patients	Severe COVID-19 confirmed by real-time RT-PCR assay; patients in respiratory distress, with RR ≥ 30 beats/min, oxygen saturation level ≤ 93% in resting state and arterial partial pressure of oxygen (PaO_2_)/fraction of inspiration O2 (FiO_2_) ≤ 300 mmHg	MSCs of non-specified origin	1 × 10^6^ MSCs/kg, i.v. infusion, single dose (*n =* 7), two doses (*n =* 7), three doses (*n =* 11)	Effectiveness, serum levels of LAC, cTnT and CK-MB were elevated significantly after MSCs therapy	Three cases experienced treatment-related side effects, specifically liver dysfunction, heart failure and allergic rash	Chen *et al*, 2020 [141]
Case report	China	1 patient	RT-PCR assay confirmed that the patient’s specimen tested positive for COVID-19, severe shortness of breath, SpO_2_ of 87.9%, computerized tomography evidences pneumonia and ground-glass opacity in bilateral lungs	Allogeneic, UC-MSC	Single dose of 1 × 10^6^ MSCs/kg, i.v. infusion	Improvement of pulmonary function and symptoms, increase of lymphocyte subsets, decrease of IL-6, TNF-α, and C-reactive protein levels, safety and efficiency	No treatment-related adverse events	Zhang et al, 2020 [142]
Pilot study	China	2 patients	Severe COVID-19; patient 1 with increased leukocyte count and neutrophils, decreased hemoglobin and lymphocytes, X-ray indicates large, patchy, high-density lesions in bilateral lungs; patient 2 with increased neutrophils and decreased leukocyte counts and lymphocytes, chest X-ray indication of patchy high-density shadows in the lower lung fields and left middle lung	Allogeneic, menstrual blood–derived MSC	Three infusions of 1 × 10^6^ MSCs/kg, i.v. infusion	MSC transplantation increases the immune indicators (including CD4 and lymphocytes) and decreases the inflammation indicators (interleukin-6 and C-reactive protein), improvement of dyspnea and lung function	No treatment-related adverse events	Tang et al, 2020 [143]
Proof of concept	Spain	13 patients	COVID-associated pneumonia requiring mechanical ventilation in the ICU	Allogeneic AT-MSC	Median number of AT-MSCs per dose was 0.98 (IQR 0.5) × 10^6^/kg Single dose (*n =* 2), two doses (*n =* 10); three doses (*n =* 1), interval of 3 days	9 patients improved clinically and 7 were extubated with a median time from the first MSC dose to extubation of 7 days. Radiological improvement in sequential X-rays was confirmed in 40% of evaluable patients. A decrease in inflammatory parameters at day 5 after infusion with a decrease in C-reactive protein in 8 patients (88%), LDH in 9 (100%), and D-dimer and ferritin in 5 of 8 evaluable patients (63%), increase in the levels of total lymphocytes was observed in responders patients (B, CD4, CD8)	No treatment-related adverse events	Sánchez-Guijo *et al,* 2020 [130]
Case series	Brazil	10 patients, *n =* 7 in cell therapy arm	COVID-associated pneumonia, presenting severe acute respiratory syndrome	MSCs of non-specified origin	Single dose of 1 × 10^6^ MSCs/kg, i.v. infusion	Improvement in symptoms, significant reduction ofchest infiltration, reduction of pro-inflammatory cytokines (TNF-α), increase of peripheral lymphocyte counts (CD4+ T cells and dendritic cells), increase of anti-inflammatory gene expression and trophic factors	No treatment-related adverse events	Mazzeo and Santos, 2020 [144]
Case report	China	1 patient	Severe COVID-19 confirmed by RT-PCR, SpO_2_ 90%, P/F 243 mmHg, pulmonary exudative lesions in bilateral lungs showed in X-ray	Convalescent plasma + UC-MSC	Three infusions of 1 × 10^6^ MSCs/kg, i.v. infusion	Restoration of lung diffusion, improvement of pulmonary function, increase of oxygenation index, PaO_2_ and absolute lymphocyte count, decline in absolute neutrophils count and IL-6	No treatment-related adverse events	Peng et al, 2020 [145]
Pilot study	China	31 patients	Severe (*n =* 23) or critical (*n =* 8) COVID-19	Allogeneic, UC-MSC	Single (*n =* 11), two (*n =* 9) or three (*n =* 11) infusions of 1 × 10^6^ MSCs/kg BW, i.v. infusion	Restoration of oxygenation, downregulation of cytokine storm, improvement of lung function. Laboratory parameters tended to improve after UC-MSC therapy compared to the status before UC-MSC therapy, including elevated lymphocyte count, decreased C-reactive protein level and procalcitonin level, decreased D-Dimer levels	No treatment-related adverse events	Guo et al, 2020 [146]
Case report	China	1 patient	Severe COVID-19, SpO_2_ of 81%, chest tightness, blood pressure of 160/91 mmHg and X-ray showing ground-glass opacity in right lung	Allogeneic, UC-MSC	Three cycles (5 × 10^7^ cells each time) with a 3-day interval, i.v. infusion	Improvement of clinical indexes and symptoms, reversal of lymphopenia (increase in counts of CD3+ T cell, CD4+ T cell and CD8+ T cell)	No treatment-related adverse events	Liang et al, 2020 [147]
Phase 1, controlled, open label	China	18 patients; *n =* 9 included in the cell therapy arm	Moderate and severe COVID-19 confirmed by RT-PCR; pneumonia was evidenced using chest radiography or CT; moderate cases were defined as fever, respiratory symptoms and confirmed pneumonia; severe cases included symptoms of shortness of breath or dyspnea after activity	Allogeneic, UC-MSC	Three cycles of i.v. infusion of allogeneic UC-MSCs (3 × 10^7^ cells each infusion) on days 0, 3, and 6	Decrease of serum IL-6 biomarker of disease progression, improvement of percentage of inspired oxygen ratio, faster absorption of lung lesions	Two patients developed transient facial flushing and fever, and one patient developed transient hypoxia at 12 h post UC-MSCs infusion	Meng et al, 2020 [148]
Case report	China	1 patient	COVID-19 confirmed by RT-PCR, acute respiratory distress, SpO_2_ 90%, computerized tomography showed ground-glass opacity in both lungs, multiple organ injury (hepatic respiratory system), immunosuppression	Allogeneic, UC-MSC	Single infusion of 1 × 10^6^ MSCs/kg BW, i.v. infusion	Patient’s SpO_2_ returned to the normal level 48 h after MSC infusion, non-invasive ventilator was successfully removed after 6 days. BUN, PCT, CRP, AST, and ALT levels decreased, leucocytes gradually recovered after 13 days	No treatment-related adverse events	Zhu *et al*, 2020 [149]
Phase 2, randomized, double-blind, placebo-controlled	China	101 patients; *n =* 65 included in the cell therapy arm; *n =* 45 control, *n =* 1 excluded	Severe COVID-19 confirmed by RT-PCR; pneumonia was evidenced using chest computed radiography (CT) imaging. Dyspnea (respiratory rate ≥ 30 times/min), oxygen saturation of 93% or lower on room air; arterial oxygen partial pressure (PaO_2_)/fraction of inspired oxygen ≤ 300 mmHg; pulmonary imaging showing that the foci progressed by > 50% in 24–48 h	Allogeneic, UC-MSCs	Three cycles of i.v. infusions of UC-MSCs (4 × 107 cells) each on days 0, 3, and 6	UC-MSC administration was safe and accelerated resolution of lung solid component lesions and improvement in the integrated reserve capability after UC-MSC administration	Adverse events reported during the study was similar in the MSC group and the placebo group. All adverse events during the observation period were judged by the site investigators and found to be unrelated to UC-MSC intervention. No deaths were observed in this trial.	Shi et al, 2021 [150]
Phase 1	Iran	11 patients	COVID-19 confirmed by RT-PCR or chest X-ray; SpO_2_/FiO_2_ ≤ 315, SOFA score between 2 and 13 point, required mechanical ventilation and/or supplemental oxygen	(UC-MSC) or (PL-MSC)	Three i.v. infusions (200 × 106 cells) every other day for a total of 600 × 106 human umbilical cord MSCs (UC-MSCs; 6 cases) or placental MSCs (PL-MSCs; 5 cases)	MSCs from a prenatal source is relatively safe, tolerable, and could rapidly improve respiratory symptoms and reduce inflammatory conditions in some critically ill COVID-19 patients	Two patients developed shivering that occurred during the initial PL-MSC infusion, which was relieved by supportive treatment in less than 1 h. This shivering did not develop again during the second and third infusions.	Hashemian et al. 2021 [151]
Pilot study	China	17 patients, *n =* 9, severe and *n =* 7 critically severe, *n =* 1 excluded	Severe COVID-19 with respiratory distress, RR ≥ 30 min^−1^, oxygen saturation ≤ 93% at rest state, oxygenation index ≤ 300 mmHg, 1 mmHg = 0.133 kPa or critically severe with respiratory failure needs mechanical ventilation Shock, combined with other organ failure, patients need ICU monitoring and treatment	Allogeneic UC-MSCs	Four i.v. infusions of (1 × 108 cells) with 1-day intervals in between	UC-MSCs was safe in severe and critically severe COVID-19 pneumonia and that administration of UC-MSCs is associated with clinical benefit and changes in inflammatory and immune populations	There were two severe adverse events (SAE) during the trial. The two SAEs were considered to have no relationship with UC-MSCs transplantation.	Feng et al. 2020 [152]
Pilot study	China	7 patients, *n =* 2 severe cases, *n =* 5 mild cases	COVID-19 confirmed by RT-PCR	MSC-derived exosomes	Nebulization of MSC-derived exosomes. The concentration of exosomes for nebulization for each patient ranged from 7.66e+0.8 to 7.00e+0.7 particles/ml based on NanoSight.	The nebulization of MSC-derived exosomes is safe and beneficial for the absorption of pulmonary lesions in mild cases of COVID-19 pneumonia and in reducing cellular residue in severe cases.	No adverse events were reported	Chu et al. 2021 [153]
Phase I double-blind, randomized, controlled	USA	28 patients, *n =* 12 included in the cell therapy arm; *n =* 12 control, *n =* 4 excluded	COVID-19 confirmed by RT-PCR, peripheral capillary oxygen saturation (SpO_2_) ≤ 94% at room air, or requiring supplemental oxygen at screening, PaO_2_/FiO_2_ ratio < 300 mmHg, Bilateral infiltrates on frontal chest radiograph or bilateral ground-glass opacities on a chest CT scan	Allogeneic UC-MSC	Two i.v. infusions of (100 ± 20 × 106)	UC-MSC treatment was safe, reduced mortality and recovery time. Inflammatory cytokines were significantly decreased in UC-MSC-treated subjects at day 6.	In the UC-MSC treatment group, the only reported adverse event occurred in a subject with bradycardia required transient vasopressor treatment.	Lanzoni et al., 2021 [154]

*MSCs*, mesenchymal stem/stromal cells; *UC-MSCs*, umbilical cord-derived mesenchymal stem cells; *AT-MSCs*, adipose tissue-derived mesenchymal stem cells; *MSCs-Exo*, mesenchymal stem/stromal cell exosomes; *ARDS*, acute respiratory distress syndrome; *BW*, body weight; *PL-MSC*, placenta-derived mesenchymal stem cells

The first SARS-CoV-2 case treated with stem cells has been reported in China [147]. A 65-year-old patient with severe pneumonia, respiratory failure, and multiorgan failure requiring mechanical ventilation was treated with three doses of umbilical cord MSCs 3 days apart, with impressive improvements in clinical and laboratory parameters a few days after MSC infusion. In a small clinical trial [66], the investigators compared seven patients (one critically severe, four severe, and two non-severe) infected with SARS-CoV-2 who received one dose of MSC therapy versus three patients in the control group (all severely ill) who received placebo. After 2–4 days, all symptoms resolved in the intervention group and no adverse events were observed. In the control group, only one patient recovered. However, both trials had limitations. The first [147] is a case report, and the patient could have improved despite cell therapy. The latter [66] is a small, poorly controlled, nonrandomized clinical trial. There is no description of how the control group was chosen, no sample size calculation, and no primary outcome, and due to the small number of patients, no conclusion was reached. In summary, the trial was powered only for hypothesis generation.

Additional case reports have described the intravenous use of umbilical cord mesenchymal stem cells (UC-MSCs; 1 × 10^6^ cells/kg), alone or with convalescent plasma, in cases of severe COVID-19. Interestingly, CD3^+^, CD4^+^, and CD8^+^ T cell counts increased after cell therapy, along with a reduction in the levels of pro-inflammatory cytokines and other markers. A recent case series of patients with COVID-19 with acute respiratory failure (*n* = 25) assessed the effect of MSCs (1 × 10^6^ cells/kg) administered in one (*n* = 7), two (*n* = 7), or three (*n* = 11) infusions. The authors reported no fatalities and found clinical and/or radiologic improvements in all cases. Laboratory markers of inflammation were not altered, but serum levels of lactate, cardiac troponin T, and creatine kinase (CK)-MB increased significantly after MSC therapy. The authors also reported side effects in three patients, including liver dysfunction, heart failure, and allergic rash [141]. The lack of data on cell origin and characterization (i.e., TF expression) does not allow detailed analysis of the results, which contrasts with previously published data (lack of evidence for immunomodulatory actions along with organ dysfunction) and could be the result of heterogeneity between the evaluated cell products.

Recently, a single-center, double-blind, phase 1/2a randomized study designed by Lanzoni et al. [154] was performed in which UC-MSCs were systemically administered to assess their safety and explore efficacy in 24 COVID-19 ARDS patients. The eligibility criteria were hospitalized patients diagnosed with COVID-19 and at least 18 years of age. Twelve patients were allocated to UC-MSC treatment, and the other 12 were allocated to the control group. Two doses of 100 ± 20 × 10^6^ UC-MSCs or vehicle solution were administered to the patients over intravenous (IV) infusion for 10 ± 5 min, on days 0 and 3 in both groups. The results of this trial indicate that UC-MSC infusions in COVID-19 patients with ARDS are safe. In terms of efficacy, 28 days after the last infusion, a significant improvement in patient survival was observed, with 91% in the UC-MSC group and 42% in the control group. The UC-MSC treatment group is characterized by a reduction in the levels of inflammatory molecules, including IFN-γ, IL-6, and TNF-α cytokines, and regulated on activation, normal T cell expressed and secreted chemokine (RANTES) in COVID-19 patients. Indeed, UC-MSC treatment was associated with a significant reduction in no serious adverse events (SAEs), mortality, and time to recovery compared with controls.

Finally, there is a possibility of developing EV-based cell-free therapies for COVID-19. In one study, patients with COVID-19 and moderate to severe ARDS received a single intravenous infusion of bone marrow MSC–derived exosomes, which was well tolerated and led to improvements in oxygenation and amelioration of inflammatory markers [140]. However, the study did not adequately report the product characterization and dose.

## Challenges, access, and therapeutic window

Several challenges must still be overcome to translate MSC-based therapies into the armamentarium of therapeutic options against COVID-19. There are challenges involved translating all methods and processes to obtain advanced therapy medicinal products, complex in nature, to reach GxP (good manufacturing/clinical/laboratory/storage/distribution/review practice) regulatory compliance. The costs of manufacturing and clinical development are considerable barriers. Product heterogeneity is a reality and may account for significantly different results, both in terms of safety and efficacy, as shown by different studies exploring similar products in terms of cell origin and minimal required characterization [156]. Therefore, there is an urgent need to identify the mechanisms of action in vivo and to further improve product characterization (e.g., through additional markers, potency assays) to increase the predictability of outcomes [157]. Moreover, to date, only small case series or case reports have been published, and although these are important for generating hypotheses, there is a need for larger randomized controlled clinical trials to generate further substantial evidence regarding the safety and effectiveness of such therapies.

Second, access and scalability should be further optimized. Due to their complexity and cost, cell-based therapies are unlikely to become first-line approaches for this disease, but might be beneficial to treat severe COVID-19 cases. With advances in MSC manufacturing technologies, the ability to produce an extensive quantity of cells for clinical use while maintaining their properties remains a challenge, but over time has become more feasible (i.e., application of 3D culture on microcarriers in bioreactors) [157]. With the need for continuous production qualification and comparability studies to mitigate the effects of donor/product heterogeneity and interbatch variability, novel strategies that utilize cell lines or iPSC-derived MSCs may contribute to increased feasibility and scalability [158]. However, product availability in real life requires extensive efforts not only in large-scale manufacturing but also in distribution and site qualification for adequate product handling until infusion. A complete logistic chain has been under development for the past few years since cell and gene therapy products have begun to reach the market [159].

Third, there is a need to not only determine safety and efficacy but also to investigate the ideal therapeutic window for cell-based therapies for severe COVID-19. The mechanisms of action of MSCs and previous preclinical and clinical experience suggest that early administration (i.e., during the first days of mechanical ventilation) might be beneficial in controlling inflammation, mitigating damage, and preventing later complications, such as lung fibrosis. This premise has been followed by most ongoing clinical trials (Table 1). It is important, however, that further studies provide data to support clinical indications, identify biomarkers to predict response, and evaluate cases of more advanced disease. Finally, although studies so far are scarce, there is a great opportunity to utilize MSC-derived exosomes as cell-free products instead of transferring living cells, which holds the potential to reduce risks and increase the predictability of outcomes.

## Conclusions

The mechanistic rationale, preliminary data, and previous results of MSC-based therapies for the treatment of ARDS and other lung diseases make them a promising therapy for severe and critical cases of COVID-19. The mechanisms of action of MSCs, which combine antiviral, immunomodulatory, and antifibrotic actions, situate these products in a position to complement the current arsenal against COVID-19 and reduce current unmet medical needs. However, large randomized controlled clinical trials are still needed to properly evaluate the safety and efficacy of each specific cell product.

The success of cell-based therapies during a global health emergency such as COVID-19 might help accelerate the development of the whole field of cell and gene therapy by increasing knowledge diffusion among clinicians and solving logistics and manufacturing challenges.

## Data Availability

Non applicable. No original data was included in this review, being comprised exclusively of published data.

## Abbreviations

SARS: Severe acute respiratory syndrome
TMPRSS2: Transmembrane protease, serine 2
IL-2R: Interleukin-2 receptor
HLA: Human leukocyte antigen
SDF-1: Stromal cell–derived factor 1
CK-MB: Creatine phosphokinase-MB
